# Target coverage and organs at risk dose in hypofractionated salvage radiotherapy after prostatectomy

**DOI:** 10.1016/j.phro.2024.100600

**Published:** 2024-06-19

**Authors:** Floor H.E. Staal, Jorinde Janssen, Sajee Krishnapillai, Johannes A. Langendijk, Stefan Both, Charlotte L. Brouwer, Shafak Aluwini

**Affiliations:** University of Groningen, University Medical Centre Groningen, Department of Radiation Oncology, Hanzeplein 1, Postbus 30.001, 9700 RB Groningen, The Netherlands

**Keywords:** Prostate bed, Salvage radiotherapy, Hypofractionation, PERYTON-trial, Planning target volume margin, Interfractional OAR volume change

## Abstract

**Background and purpose:**

Introducing moderately hypofractionated salvage radiotherapy (SRT) following prostatectomy obligates investigation of its effects on clinical target volume (CTV) coverage and organ-at-risk (OAR) doses. This study assessed interfractional volume and dose changes in OARs and CTV in moderately hypofractionated SRT and evaluated the 8-mm planning target volume (PTV) margin.

**Materials and methods:**

Twenty patients from the PERYTON-trial were included; 10 received conventional SRT (35 × 2 Gy) and 10 hypofractionated SRT (20 × 3 Gy). OARs were delineated on 539 pre-treatment Cone Beam CT (CBCT) scans to compare interfractional OAR volume changes. CTVs for the hypofractionated group were delineated on 199 CBCTs. Dose distributions with 4 and 6 mm PTV margins were generated using voxel-wise minimum robustness evaluation of the original 8-mm PTV plan, and dose changes were assessed.

**Results:**

Median volume changes for bladder and rectum were −26 % and −10 %, respectively. OAR volume changes were not significantly different between the two treatment schedules. The 8-mm PTV margin ensured optimal coverage for prostate bed and vesicle bed CTV (V95 = 100 % in >97 % fractions). However, bladder V60 <25 % was not achieved in 5 % of fractions, and rectum V60 <5 % was unmet in 33 % of fractions. A 6-mm PTV margin resulted in CTV V95 = 100 % in 92 % of fractions for prostate bed, and in 86 % for vesicle bed CTV.

**Conclusions:**

Moderately hypofractionated SRT yielded comparable OAR volume changes to conventionally fractionated SRT. Interfractional changes remained acceptable with a PTV margin of 6 mm for prostate bed and 8 mm for vesicle bed.

## Highlights

• Hypofractionation had similar interfractional changes as conventional fractionation.
• Bladder volume changed significantly, yet target coverage remained excellent.
• A planning target volume margin of 6 mm to the prostate bed seems sufficient.
• A planning target volume margin of 8 mm to the vesicle bed is needed.

## 1 Introduction

Radical prostatectomy, external beam radiotherapy (EBRT) and brachytherapy are the primary treatment options for patients with localized prostate cancer. After radical prostatectomy, up to 40% of patients experience a biochemical recurrence [1]. Salvage radiotherapy (SRT) is the only curative treatment option for patients with a biochemical recurrence after prostatectomy, with a 5-year progression-free survival up to 60% [2].

For primary prostate cancer, prostate motion has been studied with several fractionation schedules [3], [4]. However, in SRT, the clinical target volume (CTV) is the prostate bed, a region defined by anatomical landmarks that includes deformable organs at risk (OARs) [5]. As a result, interfractional volume changes of these OARs (i.e., rectum and bladder) can complicate accurate delivery of radiation dose, potentially leading to a decrease in local control and an increase in toxicity. With the use of hypofractionated SRT (i.e., a shorter treatment time with a higher dose per fraction), any shift may result in a higher risk of suboptimal target coverage and/or increased risk of delivering excessive radiation to these OARs. Therefore, in the context of hypofractionated SRT, it becomes even more important to ensure accuracy in dose delivery [6].

Current guidelines and various studies recommend a planning target volume (PTV) margin range of 5 to 15 mm for SRT, depending on the applied technique [6]. However, these studies employed diverse imaging modalities, verification techniques, preparation methods, and focused on interfractional motion, neglecting to assess volumetric and calculated dose changes [7], [8], [9], [10], [11]. More importantly, these studies did not incorporate a moderately hypofractionation schedule.

When introducing the concept of moderately hypofractionation in SRT, it is crucial to examine the impact of hypofractionation on target coverage and the dose received to OARs. Therefore, this study aimed to assess the interfractional volume and consequential dose changes of OARs and CTV during both moderately hypofractionated SRT and conventionally fractionated SRT. Furthermore, we evaluated the robustness of a range of PTV margins, varying from 4 mm to 8 mm.

## 2 Materials and methods

Cone-beam computed tomography (CBCT) images of the first 20 patients with complete daily CBCT data included in the PERYTON-trial (ClinicalTrials.gov Identifier: NCT04642027) and treated with SRT in the University Medical Centre of Groningen (UMCG) were retrospectively analysed. The PERYTON-trial, a phase III randomised controlled trial, compares moderately hypofractionated SRT with conventionally fractionated SRT. Ten patients received the conventional schedule of 35 × 2 Gy, and ten patients received the hypofractionated schedule of 20 × 3 Gy. Full inclusion and exclusion criteria of the PERYTON-trial are reported in the published trial protocol [12]. All patients provided written informed consent. A total of 577 pre-treatment CBCT images of 20 patients enrolled in the PERYTON-trial were identified between September 2020 and May 2021. Thirty-eight CBCTs were excluded for analyses because of a too short imaging field of the CBCTs to delineate the whole bladder and/or rectum. This resulted in 340 pre-treatment CBCTs from 10 patients receiving 35 × 2 Gy and 199 CBCTs from 10 patients receiving 20 × 3 Gy.

### 2.1 Treatment preparation and delivery

All patients underwent a planning-CT scan (2 mm slice thickness) with instructions to empty their bladder one hour prior to the planning-CT and were asked to drink 500 mL of water during the waiting hour. The same bladder instructions were applicable before the treatment fractions. No rectal preparation technique was used. Only if the rectum was overfilled on planning-CT (>4 m diameter) the patient received a new CT. The CTV was delineated according to the guideline of the Genito Urinary Radiation Oncologists of Canada (GUROC) group [13]. According to the PERYTON-trial and institutional protocol, an 8 mm isotropic expansion of the CTV was used to generate the PTV. For target volume coverage, ≥95% of the PTV should receive 95% of the prescribed dose (PTV V95 ≥95%) and CTV V95 = 100%. Details on the radiotherapy treatment protocol of the PERYTON-trial and the dose constraints were published earlier [5]. For verification, a CBCT was performed before each treatment and bony alignment was used; a grey-value based image registration using an image verification mask including the symphysis, sacrum, pelvis, and femoral heads.

### 2.2 Interfractional volume changes of OARs

All CBCT images were imported in Mirada RTx (Mirada Medical Ltd, Oxford) and co-registered to the planning-CTs according to the clinical treatment protocol using a bony verification mask (translations only). Bladder and rectum were delineated on all CBCTs. The percent changes in interfractional OAR volumes were calculated and compared between the conventional schedule and the hypofractionated schedule. Furthermore, the correlation between the number of fractions and interfractional volume change was analysed.

### 2.3 Evaluation of PTV margin (8 mm) for the hypofractionated SRT

One experienced physician delineated the CTV for only the hypofractionated group, as several studies have already described the CTV and OARs volume change with the conventional schedule. The CTVs were checked by another observer. To evaluate our current PTV margin (8 mm) for hypofractionated SRT, the CTV was delineated on 199 daily CBCTs of 10 patients receiving 20 × 3 Gy. The CTV was divided into two regions: the prostate bed and seminal vesicles region (Supplementary material). To evaluate interfractional dose changes, all CBCT contours (CTVs and OARs) were rigidly transferred to the planning-CT after image registration (as during treatment) and the coverage of CTV and dose constraints of the bladder and rectum were calculated. A CTV coverage constraint of V95 = 100% in >90% of all fractions was a prerequisite [12]. For OAR dose constraint evaluation, the mean dose to bladder and rectum were calculated and compliance to bladder V60 <25% and rectum V60 <5% were analysed.

### 2.4 Reducing the PTV margin

Smaller PTV margins were simulated in RayStation 11B (RaySearch Laboratories, Sweden) by using the voxel-wise minimum robustness evaluation approach [14], [15]. Three treatment plans per patient were derived from the initial treatment plan by applying a setup error of 2 mm (corresponding to PTV margin of 6 mm) and 4 mm (corresponding to PTV margin of 4 mm) in 14 directions and constructing the voxelwise minimum dose distribution. All dose parameters of the CBCT contours were calculated.

### 2.5 Statistics

The mean interfractional volume change was calculated per patient. The median of these mean volume changes per patient was used to compare the conventional versus the hypofractionated schedule, using the Mann Whitney-*U* test. Pearson’s R coefficient was used to assess any correlation with the number of fractions. Statistical analyses were performed using Statistical Package for the Social Sciences (SPSS). Released 2021. IBM SPSS Statistics for Windows, Version 28.0. Armonk, NY: IBM Corp.

## 3 Results

### 3.1 Interfractional volume changes of OARs

The median volumes of the bladder and rectum on planning-CT were 337 cm^3^ (IQR 197 cm^3^ to 503 cm^3^) and 130 cm^3^ (IQR 83 cm^3^ to 157 cm^3^), respectively (Table 1). Median percent change in bladder and rectum volumes were −25.7 (interquartile range (IQR) of −43.8 to −5.7) and −10.4 (IQR of −25.2 to 13.8), respectively. No significant differences in bladder and rectum volume changes were observed between the conventional versus the hypofractionated schedule (Table 1).

**Table 1:** Interfractional percent volume changes of bladder and rectum relative to the planning computed tomography (CT) scan.

	Total		Conventional fractionated SRT* (35 × 2 Gy)		Hypofractionated SRT* (20 × 3 Gy)		
	(N = 20: 539 CBCTs*)		(N = 10: 340 CBCTs*)		(N = 10: 199 CBCTs*)		
**Bladder**	**Median**	Q1* to Q3*	**Median**	Q1* to Q3*	**Median**	Q1* to Q3*	P**
Volume planning-CT (cm^3^)	346.3	197.0 to 503.4	297.3	177.6 to 384.6	395.2	240.6 to 534.8	0.17
Volume change CBCT * (%)	−25.7	−43.8 to −5.7	−18.8	−45.3 to 8.8	−26.8	−43.0 to −17.2	0.55

**Rectum**							
Volume planning-CT (cm^3^)	130.9	83.0–156.8	136.0	83.9 to 154.0	121.5	76.8 to 160.4	0.94
Volume change CBCT * (%)	−10.4	−25.2 to 13.8	−6.0	−26.7 to 21.6	−10.4	−19.2 to 10.5	0.76

*SRT: salvage radiotherapy; CBCT: Cone beam computed tomography; Q1: first quartile; Q3: third quartile; SD: standard deviation; **Mann Whitney U was performed.

**Fig. 1:**
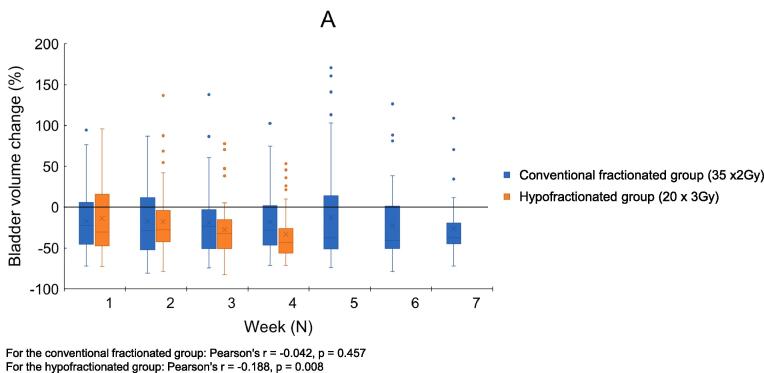

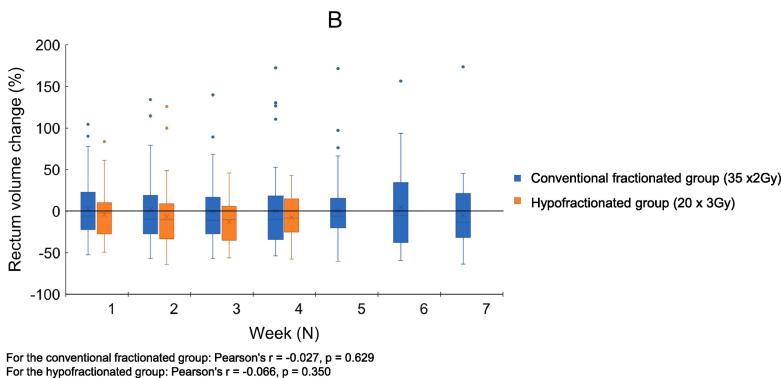
Interfractional volume change of bladder (A) and rectum (B) of 10 patients receiving conventional (35 × 2 Gy) versus 10 patients receiving hypofractionated (20 × 3 Gy) salvage radiotherapy after prostatectomy.

In the conventional fractionated group, no correlation was seen between the number of fractions given and bladder and rectum volume change (Fig. 1). Furthermore, in the hypofractionated group, only bladder volume correlated with the number of treatment fractions, showing a decrease in volume throughout the treatment course (Pearson's r = −0.19, p = 0.01).

### 3.2 Evaluation of PTV margin (8 mm) for the hypofractionated SRT

The median volume of the prostate bed CTV on planning-CT was 86 cm^3^ (range: 54 cm^3^ to 117 cm^3^). Overall, the prostate bed volume on CBCT was similar to the planning-CT volume, with a median percent change of 2.3% (IQR of −11 to −0.1) (Table 2).

**Table 2:** Interfractional percent volume and dose changes in 10 patients treated with hypofractionated salvage radiotherapy after prostatectomy (20 × 3 Gy).

	N = 10, 199 CBCTs*	
	**Median**	Q1* to Q3*
Prostate bed volume (%)**	−2.3	−11.0 to −0.1
Prostate bed CTV V95%*	100.0	100.0 to 100.0
Vesicle bed volume (%)**	−5.6	−19.0 to −1.0
Vesicle bed CTV V95%*	100.0	100.0 to 100.0
Bladder mean dose (%)	29.5	22.5 to 38.6
Bladder V60 (%)*	9.7	7.2 to 16.9
Rectum mean dose (%)	24.7	21.7 to 31.0
Rectum V60 (%)*	3.9	2.9 to 5.3

*CBCT: cone beam computed tomography scan; CTV: clinical target volume; CTV V95: the CTV volume percentage receiving ≥95 % of the prescribed dose; V60: volume percentage receiving 60 Gy or more; Q1: first quartile; Q3: third quartile **Expressed relative to percentage of planning scan.

**Fig. 2:**
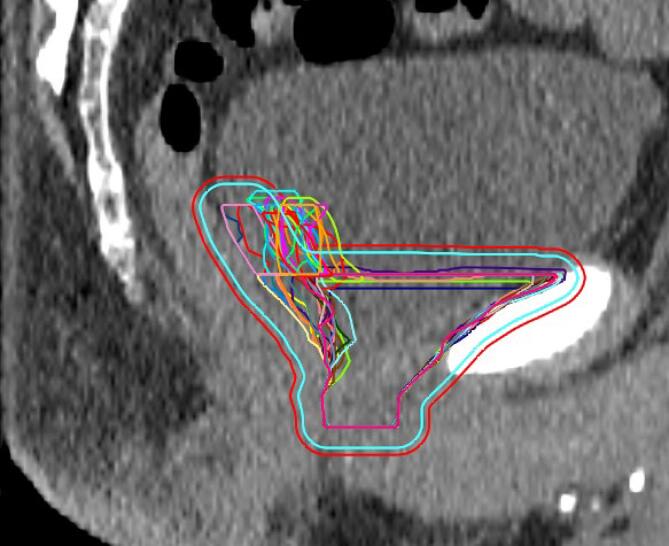
Example of all interfractional clinical target volumes (CTVs) within one patient during moderately hypofractionated salvage radiotherapy after prostatectomy (20 × 3 Gy), with a planning target volume (PTV) margin of 8 mm (red contour) and a PTV margin of 6 mm (light blue).

The median volume of the seminal vesicles bed CTV on planning-CT was 52 cm^3^ (range: 14 cm^3^ to 100 cm^3^), with a median percent change on CBCT of −5.6% (IQR of −19.0 to −1.0)). No correlation was observed between the number of treatment fractions given and the prostate bed CTV volume (Pearson's r = 0.05, p = 0.47), nor with the seminal vesicles bed CTV volume (Pearson's r = −0.03, p = 0.70). All treatment plans fulfilled the coverage constraints PTV V95 ≥95% and CTV V95 ≥99%. Among the 199 CBCTs, the use of PTV margin of 8 mm resulted in an excellent target coverage, with prostate bed CTV V95 = 100% in all treatment fractions, and only 6/199 fractions (3%) failed to meet the CTV V95 = 100% of the vesicle bed (range 98 to 100%) (Fig. 2).

Regarding the OARs dose constraints, all treatment plans met the bladder V60 <25% constraint, and only 1/10 treatment plans failed to meet rectum V60 <5%. During treatment, 5% (10/199) of fractions failed to meet bladder V60 <25% and 33% (65/199) failed to meet rectum V60 <5%. Of note, bladder V60 was <30% in 99% (197/199 fractions) and rectum V60 was <10% in 24% (47/199 fractions). As expected, small bladder volume was associated with a higher mean bladder dose (Pearson’s r = −0.72, p < 0.01), and small rectum volume with a higher mean rectal dose (Pearson’s r = −0.38, p < 0.01).

### 3.3 Reducing the PTV margin

**Fig. 3:**
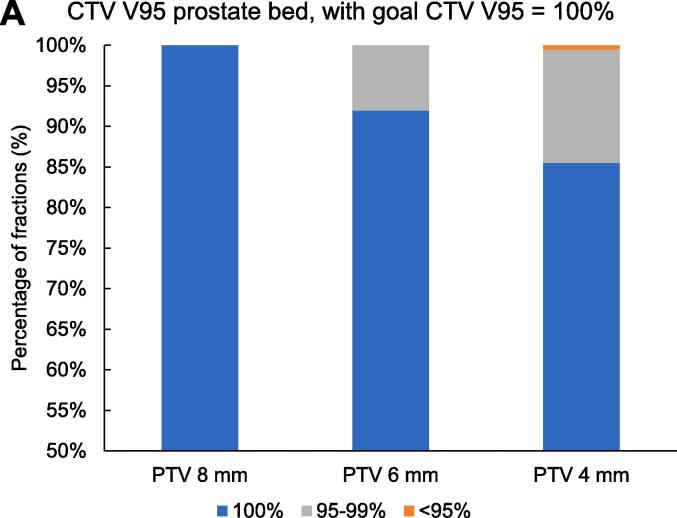

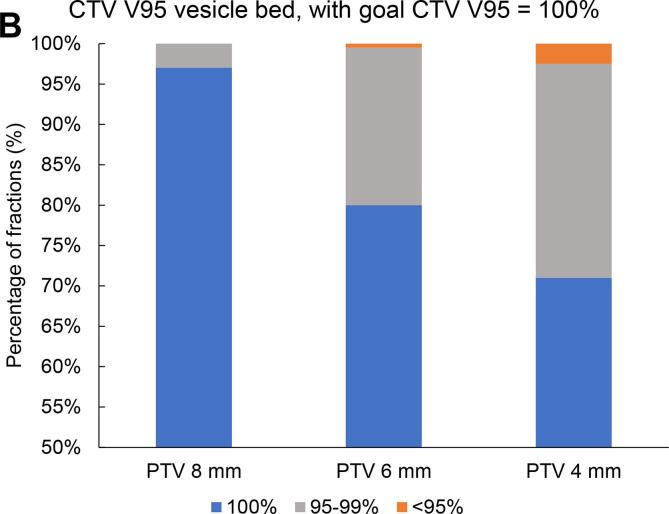
Frequency distribution of interfractional dosimetry of 10 patients receiving hypofractionated (20 × 3 Gy) salvage radiotherapy (A) clinical target volume (CTV) V95 of the prostate bed, and (B) CTV V95 of the seminal vesicles bed, derived from the initial treatment plan (planning target volume margin ((PTV) of 8 mm) and the simulated treatment plans with a PTV margin of 8 mm, 6 mm and 4 mm.

**Fig. 4:**
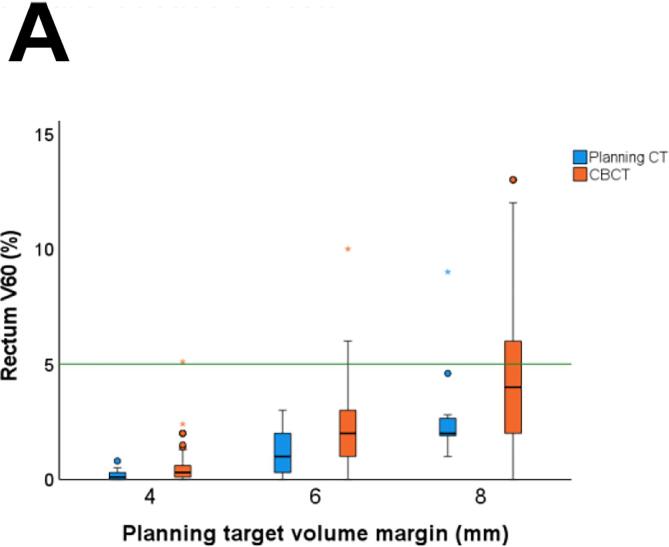

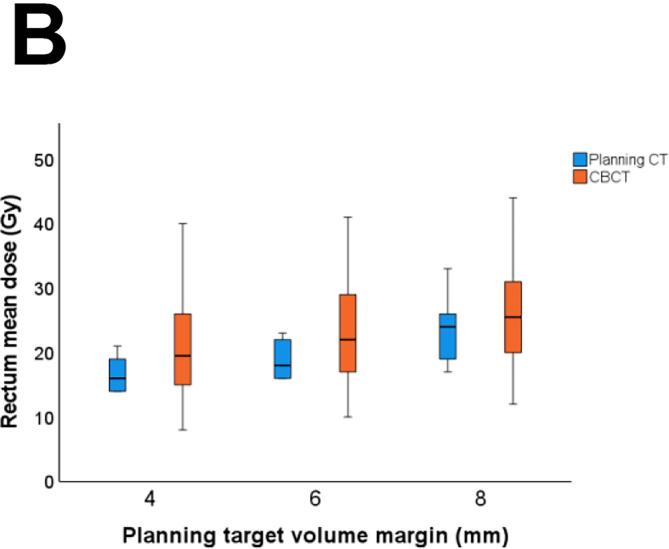

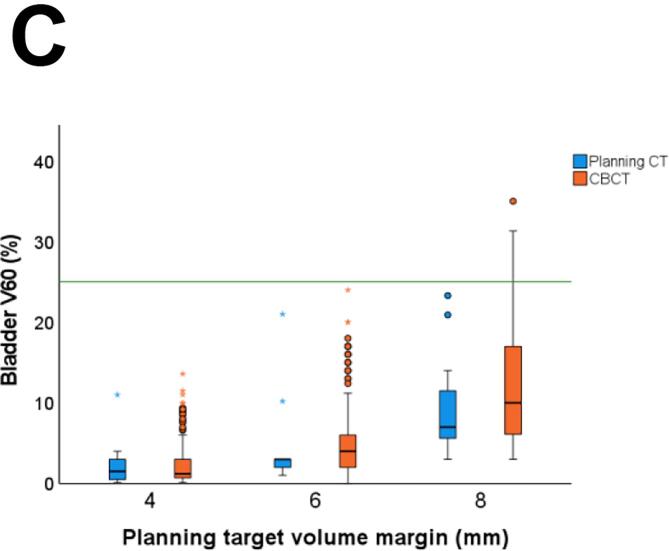

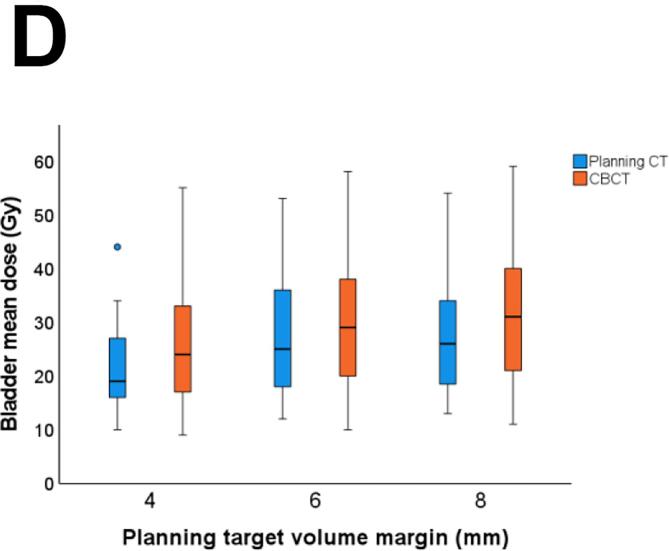
Interfractional dose change of the organs at risk (OARs) of the treatment plan and on cone beam computed tomography (CBCT) scan per treatment plan: the original plan (planning target volume (PTV) margin of 8 mm) and the derived treatment plans with a PTV margin of 6 mm and 4 mm. (A) Rectum V60 (B) Mean rectum dose, (C) Bladder V60, and (D) Mean bladder dose.

With a reduced PTV margin of 6 mm, the predefined CTV coverage constraint of V95 = 100% in >90% of all fractions was met for the prostate bed CTV (CTV V95 = 100% in 92% (183/199 fractions)) and in only 86% (171/199) for the seminal vesicles bed CTV V95 = 100% (Fig. 3). With a 6 mm PTV margin, all fractions met the bladder V60 <25% dose constraint, and 97% (194/199 fractions) met the rectum V60 <5% dose constraint (Fig. 4). Reducing the PTV margin to 4 mm, prostate bed CTV V95 = 100% was met in only 74% (147/199 fractions). With a 4 mm PTV margin, CTV coverage decreased with larger bladder volume change and rectum volume change (Pearson’s r= −0.48, p<0.001, and Pearson’s r = 0.19, p= 0.011, respectively). With PTV margin 6 and 8 mm, we did not see any correlation between CTV coverage and OARs volume change.

## 4 Discussion

This study evaluated the interfractional volume and dose changes in the first 20 patients treated within the PERYTON-trial, a phase III RCT investigating the role of moderately hypofractionated prostate bed salvage radiotherapy after prostatectomy (20 × 3 Gy) [12]. Despite the shorter treatment regimen, the rectum and bladder volume changes in the hypofractionated group were similar to those observed in the conventionally fractionated group (35 × 2 Gy). Our results confirm that the application of our current protocol, including an 8-mm PTV margin along with patient bladder preparation, ensure an excellent target coverage while avoiding excessive radiation dose to OARs in hypofractionated SRT. Furthermore, a reduction of the PTV to 6 mm for the prostate bed CTV resulted in sufficient target coverage, with 95% of all treatment fractions meeting CTV V95 = 100%, supporting the use of differential margins at the prostate bed and respectively at seminal vesicles CTV level.

Despite employing a bladder preparation protocol, we observed large bladder volume changes (median of −26%). A study on conventional SRT, using the same bladder preparation, reported similar bladder volume change (mean of −33%), which highlight that the current bladder preparation does not ensure stable bladder filling [16]. Furthermore, we noted a correlation between large bladder volume change with inadequate CTV coverage. This aligns with a study on conventional fractionated SRT, involving 377 CBCTs of 40 patients, which found that bladder size change led to geographic miss [17]. These results emphasize the importance of maintaining a constant bladder volume for optimal SRT outcome. Moreover, change in bladder volume may not only affect target coverage, but our results also showed that bladder volume change can lead to increased bladder dose, subsequently raising the risk of toxicity [10], [18], [19], [20], [21], [22]. Several studies have explored alternative bladder preparation techniques and ultrasound scanning before treatment, all concluded that the current methods offer limited reproducibility of bladder filling [6], [16], [17], [22], [23], [24]. As such, a universally effective bladder filling protocol remains elusive. Therefore, image-guided SRT or adaptive SRT is needed to compensate for variations in bladder filling [25], [26].

While we did not use any rectal preparation protocol, we observed a relatively small rectal volume change (median of −10%), compared to studies utilizing strict rectal preparations [16], [24], [27], [28]. For instance, a study on 18 patients undergoing ultra-hypofractionated SRT (5 fractions) instructed patients to use an enema the night before and the morning of each fraction. Surprisingly, they observed larger rectal volume change (median of +21%) [27]. A prospective study on the use of antiflatulent medication in 78 patients, found no difference in rectal size nor toxicity [28]. Furthermore, a study on 314 CBCT from 14 patients showed that using endorectal balloon (N = 8) resulted in higher rectal wall dose compared to patients treated without endorectal balloon (N = 6) [24]. Another study reported stable rectal volumes during moderately hypofractionated (20 fractions) radiotherapy to the prostate without any rectal preparation [29]. Altogether, these results suggests that strict (and sometimes invasive) rectal preparations may not be a prerequisite to maintain a stable rectal volume during SRT and may work counterproductively [24].

We observed that a 6 mm PTV margin for the prostate bed and 8 mm for the vesicle bed ensured sufficient target coverage, which aligns with prior studies on conventional SRT reporting PTV margins of 6 to 8 mm [5], [7], [30]. However, we observed insufficient vesicle bed CTV coverage with a PTV margin of <8 mm. This difference between the upper part of the CTV (i.e., vesicle bed) versus the lower part of the CTV (i.e., prostate bed) may be due to larger motion of the vesicle bed, which has previously been reported [8], [10], [22]. Therefore, our results support the use of a PTV margin of 6 mm for the prostate bed CTV and of 8 mm for the vesicle bed CTV. However, it is important to note that while interfractional CTV motion accounts for the largest component of observed geometric uncertainties, institutional variability (such as different verification methods) introduces another component of uncertainty in radiotherapy planning. As such, a comprehensive margin calculation should consider all institution-specific uncertainties.

Given that a PTV margin of 4 mm failed to ensure acceptable CTV coverage, further reducing the PTV margin seemed unfeasible. In contrast, a recent study on 14 patients treated with SRT in 35 fractions and daily CBCT, concluded that a ≤ 2 mm PTV margin ensured sufficient coverage [31]. This difference could be explained by the fact that their coverage constraint was CTV V95 >95%, while we used a constraint of CTV V95 = 100%. Moreover, a phase II trial on 18 patients treated with ultra-hypofractionated SRT (in 5 fractions) and a 5-mm PTV margin, observed that CTV V95% >93% was achieved in only 70% of fractions, indicating that a PTV margin of <6 mm seems insufficient to ensure clinically acceptable CTV coverage [27].

Other studies have explored the possibility of the application of PTV margins ≤5 mm by using methods such as surgical clips or fiducials [6], [9], [11], [32], [33], [34], [35]. However, nowadays, many surgical clips are nonradiopaque. Additionally, markers necessitate invasive procedures and carry the risk of migration. Recent studies have also reported the possibility to reduce the PTV margin with ultra hypofractionated MR-guided SRT or adaptive SRT [25], [26], [36]. However, as adaptive nor MR-guided radiotherapy is not (yet) universally available, these results are not applicable across all settings and there is a lack of data on their impact on clinical outcomes. Moreover, it is important to note that ultra hypofractionated SRT remains experimental. Therefore, our current study offers a more representative perspective of daily clinical practice.

The current study has a few limitations. First, our study did not address intrafractional changes. Several studies addressed this issue and reported very small intrafractional CTV motion (mean of 0.4–1.5 mm) making this intrafractional component less relevant [11], [20], [37]. Secondly, the soft-tissue resolution of CBCTs is sub optimal, potentially affecting delineation accuracy. To minimize inter-observer delineation variability, all contours were delineated by a single observer, and scans with poor image quality (n = 38) were excluded for this analysis. While the current study is one of the largest addressing interfractional changes during SRT, it's important to recognize the limitation of a small sample size.

In conclusion, our results showed that moderately hypofractionated salvage radiotherapy after prostatectomy yielded comparable OAR volume changes compared to conventionally fractionated SRT and underscore the feasibility of moderately hypofractionated SRT. Furthermore, interfractional changes observed were well covered within a PTV margin of 6 mm for prostate bed and 8 mm for vesicle bed. While challenges persist in the ongoing pursuit of more precise motion assessment, our study presents a perspective aligned with daily clinical practice.

## Funding statement

The PERYTON-trial is sponsored by Dutch Cancer Society (grant number 12649).

## CRediT authorship contribution statement

**Floor H.E. Staal:** Conceptualization, Methodology, Formal analysis, Investigation, Data curation, Writing – original draft, Visualization. **Jorinde Janssen:** Conceptualization, Methodology, Writing – review & editing. **Sajee Krishnapillai:** Investigation, Writing – review & editing. **Johannes A. Langendijk:** Writing – review & editing, Supervision. **Stefan Both:** Writing – review & editing. **Charlotte L. Brouwer:** Conceptualization, Methodology, Resources, Writing – review & editing, Supervision. **Shafak Aluwini:** Conceptualization, Methodology, Resources, Writing – original draft, Writing – review & editing, Supervision.

## Declaration of competing interest

The authors declare the following financial interests/personal relationships which may be considered as potential competing interests: The PERYTON-trial is sponsored by Dutch Cancer Society (KWF Kanker Bestrijding grant number 12649). S. Aluwini reports a role as Chair of the LPRU (Landelijk Platform Radiotherapie Urologische Tumoren). J.A. Langendijk reports a relationship with Dutch Cancer Society that includes: funding grants. J.A. Langendijk reports a relationship with Global Scientific Advisory Board of IBA. That includes: board membership. J.A. Langendijk reports a relationship with RayCare International Advisory Board of RaySearch that includes: board membership. J.A. Langendijk reports a relationship with IBA that includes: speaking and lecture fees. J.A. Langendijk reports a relationship with the Netherlands Society for Radiation Oncology that includes: board membership.

## Data Availability

Research data are stored in an institutional repository and will be shared upon request to the corresponding author.
