# Supplementary figures and images for: Target coverage and organs at risk dose in hypofractionated salvage radiotherapy after prostatectomy
Source: Phys Imaging Radiat Oncol. 2024 Jun 19;31:100600. doi: 10.1016/j.phro.2024.100600 (PMC11254181; doi:10.1016/j.phro.2024.100600)

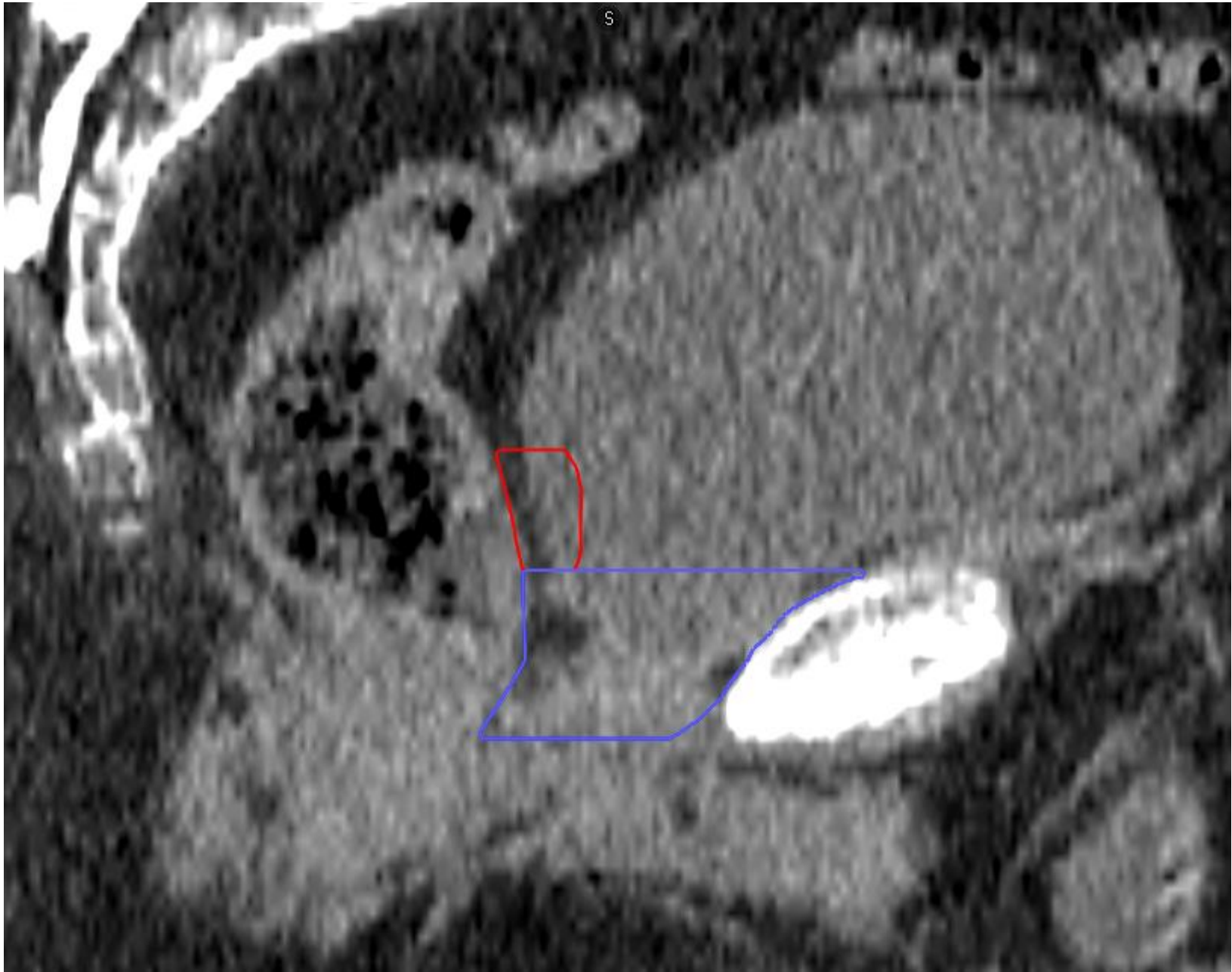

Supplement: Supplementary Data 1 [file mmc1.pdf]
